# Comparative outcomes of trans-arterial radioembolization in patients with non-alcoholic steatohepatitis/non-alcoholic fatty liver disease-induced HCC: a retrospective analysis

**DOI:** 10.1007/s00261-024-04295-8

**Published:** 2024-05-06

**Authors:** Christopher Brunson, Lucas Struycken, David Schaub, Jacob Ref, Daniel Goldberg, Jack Hannallah, Gregory Woodhead, Shamar Young

**Affiliations:** 1 Present Address: University of California, 505 Parnassus Avenue, M-391, San Francisco, CA 94143-0628 USA; 2 Department of Medical Imaging, University of Arizona Health Sciences, Tucson, USA; 3 University of Arizona Health Sciences, Tucson, USA

**Keywords:** Hepatocellular carcinoma, Radiation dosimetry, Yttrium-90, Fatty liver disease

## Abstract

**Purpose:**

Tumorigenesis in NAFLD/NASH-induced HCC is unique and may affect the effectiveness of trans-arterial radioembolization in this population. The purpose of this study was to retrospectively compare the effectiveness of trans-arterial radioembolization for the treatment of hepatocellular carcinoma (HCC) between patients with non-alcoholic steatohepatitis (NASH)/non-alcoholic fatty liver disease (NAFLD) and non-NASH/NAFLD liver disease.

**Materials and methods:**

Consecutive patients with HCC who underwent TARE at a single academic institution were retrospectively reviewed. Outcome measures including overall survival (OS), local progression-free survival (PFS), and hepatic PFS as assessed by modified response evaluation criteria in solid tumors (mRECIST) were recorded. Kaplan–Meier and Cox proportional hazard models were utilized to compare progression-free survival and overall survival.

**Results:**

138 separate HCCs in patients treated with TARE between July 2013 and July 2022 were retrospectively identified. Etiologies of HCC included NASH/NAFLD (30/122, 22%), HCV (52/122, 43%), alcoholic liver disease (25/122, 21%), and combined ALD/HCV (14/122, 11%). NASH/NAFLD patients demonstrated a significantly higher incidence of type 2 diabetes mellitus (*p* < 0.0001). There was no significant difference in overall survival (*p* = 0.928), local progression-free survival (*p* = 0.339), or hepatic progression-free survival between the cohorts (*p* = 0.946) by log-rank analysis. When NASH/NAFLD patients were compared to all combined non-NASH/NAFLD patients, there was no significant difference in OS (HR 1.1, 95% C.I. 0.32–3.79, *p* = 0.886), local PFS (HR 1.2, 95% C.I. 0.58–2.44, *p* = 0.639), or hepatic PFS (HR 1.3, 95% C.I. 0.52–3.16, *p* = 0.595) by log-rank analysis.

**Conclusion:**

TARE appears to be an equally effective treatment for NASH/NAFLD-induced HCC when compared to other causes of HCC. Further studies in a larger cohort with additional subgroup analyses are warranted.

**Graphical abstract:**

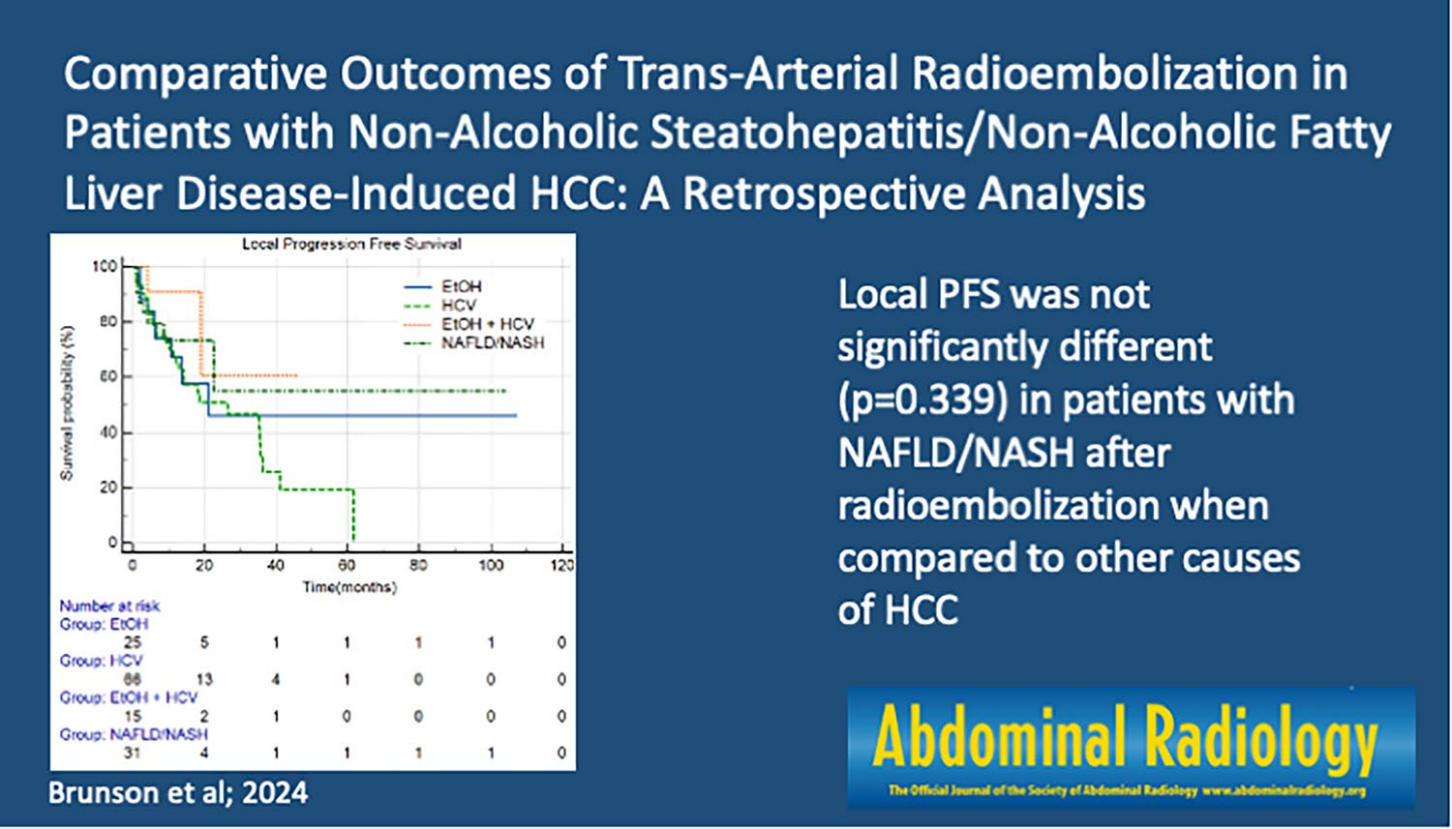

## Introduction

HCC, or hepatocellular carcinoma, is the third most common cause of cancer-related mortality world-wide [1]. Trans-arterial chemoembolization (TACE) has been historically utilized by interventional radiologists to treat patients with unresectable, liver dominant HCC; indeed, there is a preponderance of literature regarding its efficacy in this population [2]. Recently, trans-arterial radioembolization (TARE) with Yttrium-90 (Y90) microspheres has been increasingly adopted as an alternative trans-arterial treatment for HCC [3]. However, given the recency of the emergence of TARE, selecting which trans-arterial therapy to use in each patient is largely institutional and practitioner dependent [4, 5]. Because of this, data regarding the impact that patient factors such as the etiology of HCC may have on the efficacy of TARE are lacking.

Historically, hepatitis C virus (HCV)-induced liver disease and alcoholic liver disease (ALD) both comprised the leading causes of HCC in the Western hemisphere [6]. However, non-alcoholic steatohepatitis (NASH) secondary to non-alcoholic fatty liver disease (NAFLD) is now the leading cause of cirrhosis and liver transplantation in Western countries [7], surpassing HCV and ALD [8]. Because of this trend, NASH-induced hepatocellular carcinoma (HCC) is on the rise as well [9]. While NASH/NAFLD-induced HCC has been under-studied compared to HCV/ALD-induced HCC, it has gained increasing attention because of its unique neoplastic process and its propensity to affect women and under-represented minorities [10, 11]. Moreover, patients with fatty liver-induced HCC often present with larger, more advanced tumors when compared to patients with other etiologies of HCC [12], and HCC can indeed present in patients with NAFLD who do not have evidence of cirrhosis or fibrosis [13, 14]. These underlying biological differences in HCCs in patients with NASH/NAFLD suggest the inherent possibility of different clinical outcomes in this population when treated with trans-arterial radioembolization [15].

Available data regarding the outcomes of combined locoregional treatment of HCCs in patients with NASH/NALFLD are differential, with some data suggesting better outcomes for patients with hepatitis C virus (HCV) or alcohol-induced liver disease (ALD) [16] compared to NASH/NAFLD-induced HCC, and other data suggesting the opposite [17]. However, data exclusively studying outcomes of NASH/NAFLD-induced HCC treated with TARE are lacking. Given the nationwide increase in fatty liver-induced HCC and the simultaneously increasing utilization of TARE as first-line locoregional therapy in the treatment of HCC, further investigations into the efficacy of TARE in the treatment of NASH/NAFLD-induced HCC are of importance. This single-center retrospective study aims to address this need in the literature by comparing TARE outcomes according to the etiology of HCC with a focus on NASH/NAFLD-induced HCC.

## Methods

### Patient population

**Fig. 1:**
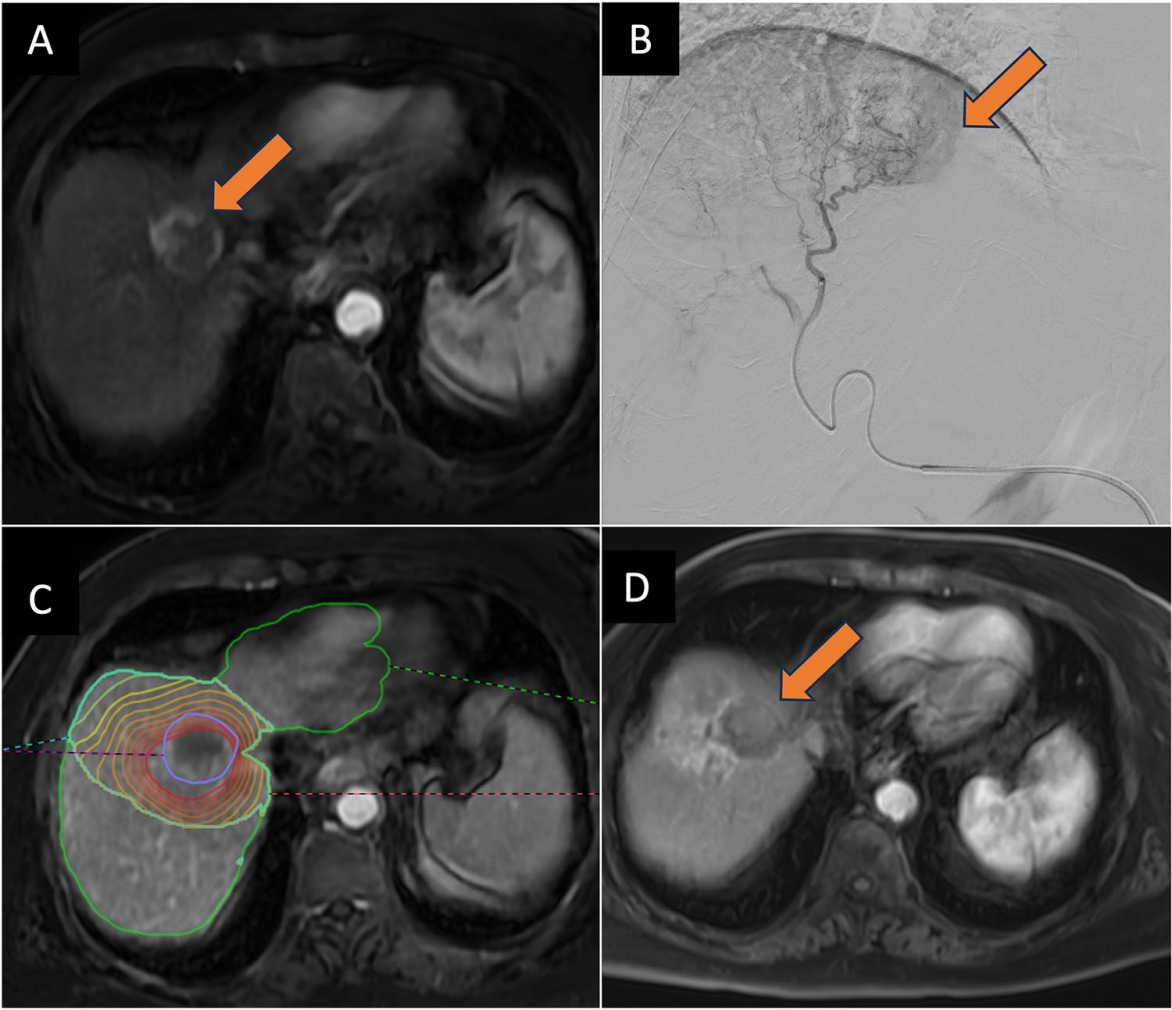
**a** Axial arterial phase magnetic resonance image (MRI) of the abdomen, demonstrating a segment 8 HCC (orange arrow) **b** Digital subtraction angiogram during planning angiography with the tip of the microcatheter positioned within the proximal segment 8 artery. Tumor enhancement is seen distal to the catheter as demonstrated by the orange arrow. **c** Post-Y90 delivery dose contour map generated using Simplicit90Y image-fusion and dosimetry planning software **d** Three-month follow-up axial arterial phase MRI of the treated tumor, demonstrating a complete response to radioembolization with adjacent post-radiation changes (Color figure online)

This single-center retrospective study was approved by the local Institutional Review Board. Patient consent was waived per the local Institutional Review Board due to the retrospective nature of the study. Patients were considered eligible for inclusion if they were adults > 18 years and underwent TARE for either biopsy-proven HCC or LIRADS-5 lesions on contrast-enhanced magnetic resonance (MR, Fig. 1a) or computed tomography (CT) imaging between 2013 and 2022 at the recommendation of a multidisciplinary tumor board. Per local tumor board policy, patients were eligible for TARE if their ECOG score was less than 2 and they did not have late-stage HCC (BCLC C). Patients were excluded from undergoing TARE if their tumors were amenable to percutaneous ablation or if they were eligible for transplant or resection. Patients were excluded from this study if there was no imaging or clinical follow-up after TARE.

### HCC case definitions

The etiology of HCC was determined by a hepatologist; specifically, patients were diagnosed with NASH/NAFLD through percutaneous biopsy or through a combination of imaging and laboratory values as is standard [18]. Patients with cryptogenic cirrhosis, primary biliary cirrhosis, hepatitis B cirrhosis, or cirrhosis secondary to hereditary hemochromatosis were excluded from analysis due to a paucity of these disease processes in the local population.

### TARE treatment

All patients underwent either lobar or segmental treatments with either glass or resin microspheres at the discretion of the performing interventional radiologist. All performing interventional radiologists had between one and nine years of experience. Conventional mapping angiography technique (Fig. 1b) as previously described was used prior to microsphere deposition [19]. Dosing targets and dosimetry methods (Fig. 1c) were at the discretion of the treating interventional radiologist and varied during the study period in accordance with newly reported dosimetry results [20, 21]. Patients received repeat treatments if necessary, according to tumor response at follow-up and multidisciplinary tumor board consensus.

### Laboratory and clinical data

Patients who met inclusion criteria had all clinical and laboratory data, including history of hypertension, diabetes, and pre-treatment AFP levels retrospectively collected. Liver function tests (LFTs) were also recorded before and after treatment.

### Outcomes data

Local progression-free survival (PFS) was defined as the time from initial treatment to first progression of the treated tumor according to modified Response Evaluation Criteria in Solid Tumors (mRECIST) criteria or throughout the liver (hepatic PFS) as assessed on follow-up contrast-enhanced CT or MR [22] (Fig. 1d). Overall survival was considered to be from the time of treatment to death and patients were censored for transplant.

### Statistical analysis

Categorical and continuous variables regarding the patient population, including demographics, were analyzed by Chi-square and analysis of variance (ANOVA) tests, respectively. Progression-free survival and overall survival were analyzed using the Kaplan–Meier method. Cox proportional hazard models were used to perform multivariate analysis on any variables with a *p* value < 0.1 on univariate analysis. Any p value less than 0.05 was considered significant. To further evaluate the role that NAFLD/NASH had on outcomes, patients were separated into a NAFLD/NASH cohort and non-NAFLD/NASH cohort and subsequently compared. Statistical analysis was performed using MedCalc statistical software (MedCalc Software ltd, Ostend, Belgium).

## Results

### Patient characteristics

A total of 195 unique HCCs were retrospectively identified. After exclusion for loss to clinical follow-up, lack of post-procedural cross-sectional imaging, and the diagnosis of hepatitis B virus-induced cirrhosis, cryptogenic cirrhosis, or hemochromatosis, 138 separate HCCs in 122 unique patients were included. Demographic data are found in Table 1. Mean age at time of treatment was 64.6 ± 8.7 years. Twenty-nine (29/122, 24%) patients were female, and 93 (93/122, 76%) were male. Thirty (30/122, 22%) patients were diagnosed with NALFD/NASH, 52 (52/122, 43%) had HCV, 25 (25/122, 21%) had ALD, and 14 (14/122, 11%) presented in patients with mixed HCV and ALD-induced cirrhosis. When comparing among the cohorts, there was a significant difference in the model for end stage liver disease-sodium (MELD-Na) score (*p* = 0.036) among the various etiologies of HCC (Table 1). A subgroup analysis comparing patients with NASH/NAFLD to non-NASH/NAFLD patients demonstrated a significantly higher incidence of type 2 diabetes mellitus (*p* < 0.001) in the NASH/NAFLD cohort. Median follow-up time was 8.5 months in the ALD cohort, 9.6 months in the HCV cohort, 13.5 months in the mixed ALD/HCV cohort, and 8.9 months in the NAFLD/NASH cohort.

**Table 1:** Demographic and clinical factors of the patient cohort at the time of radioembolization

	Entire cohort	NALFD/NASH	ALD	HCV	HCV + ALD	*P* value
Number (%)	total (n = 122)	31(25)	25(21)	52 (43)	14 (11)	–
Age, years (mean ± SD)	66 ± 8.4	71.4 ± 9.3	62.8 ± 7.5	62.6 ± 8.5	65.6 ± 4.9	0.0004
DM	total (n = 52)	27 (87)	6 (12)	16 (64)	3 (21)	0.001
Child–Pugh Score						
A	85 (70)	22 (71)	13 (52)	49 (94)	7 (50)	
B	34 (28)	7 (23)	12 (48)	3 (6)	6 (43)	
NR	3 (2)	2 (6)	0	0	1 (7)	0.016
MELD	9.7 ± 0.7	9.5 ± 3	9.9 ± 2.6	8.9 ± 2.5	10.5 ± 3.6	0.153
MELD-Na	10.3 ± 1.1	9.8 ± 3.6	11.5 ± 4.2	9.1 ± 3.4	10.8 ± 3.8	0.036
Albumin	3.6 ± 0.2	3.6 ± 0.5	3.3 ± 0.6	3.7 ± 0.7	3.6 ± 0.6	0.366
INR	1.19 ± 0.17	1.2 ± 0.2	1.2 ± 0.1	1.2 ± 0.2	1.2 ± 0.1	0.552
AST	60.6 ± 55.1	63.5 ± 90.9	48.9 ± 15.1	65.2 ± 45.2	56.2 ± 27.6	0.63
ALT	43.8 ± 42	44.6 ± 56.6	32.6 ± 13.3	50.8 ± 42.9	33.9 ± 16.4	0.234
Total Bilirubin	1.1 ± 0.6	1.1 ± 0.6	1.3 ± 0.7	1 ± 0.6	1.1 ± 0.6	0.174
Creatinine	0.9 ± 0.5	1 ± 0.3	0.9 ± 0.3	0.8 ± 0.2	1.1 ± 1.4	0.282
AFP	12.7	8.1 ± 10.1	13.9 ± 113.3	29.5 ± 119.1	27.4 ± 279.7	0.099
Tumor size, cm (mean ± SD)	4.1 ± 0.92	4.9 ± 3.2	3.6 ± 2.5	4.9 ± 4.4	3.1 ± 1.9	0.05

Reported p values are for the entire cohort

*NAFLD/NASH* (non-alcoholic fatty liver disease/non-alcoholic steatohepatitis), *ALD* (alcoholic liver disease), *HCV* (hepatitis C virus), *HCV + ALD* (hepatitis C virus + alcoholic liver disease), *SD* (standard deviation), *DM* (diabetes mellitus). Unless otherwise stated, continuous values are represented as median ± interquartile range

### TARE outcomes (overall survival and progression-free survival)

**Fig. 2:**
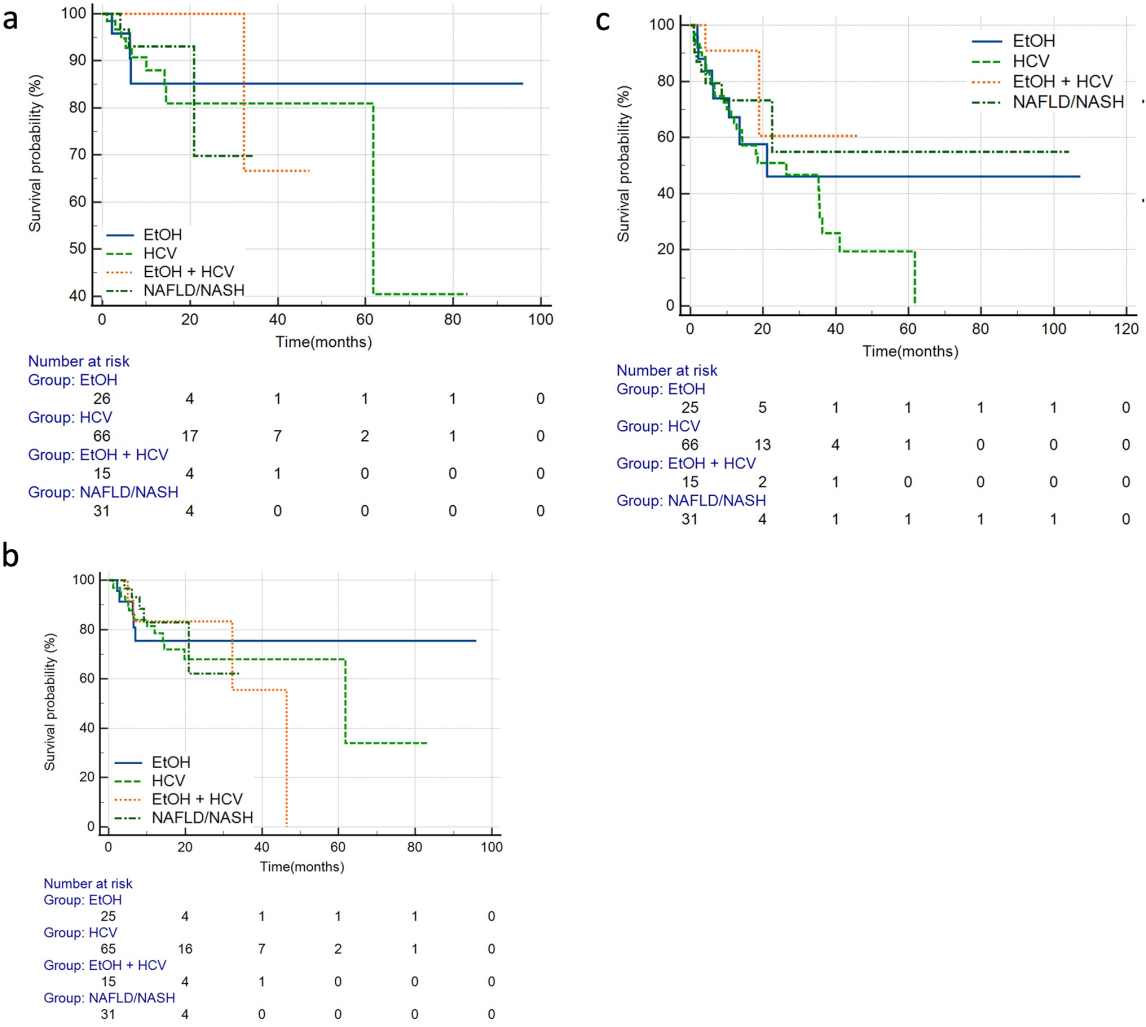
Kaplan Meier survival curves for the entire cohort **a** Overall survival (*p* = 0.928) **b** Hepatic progression-free survival (*p* = 0.946) **c** Local progression-free survival (*p* = 0.339). Abbreviations: *NAFLD/NASH* non-alcoholic fatty liver disease/non-alcoholic steatohepatitis, *ALD* alcoholic liver disease, *HCV* hepatitis C virus, *HCV + ALD* hepatitis C virus + alcoholic liver disease

For the entire cohort, Kaplan–Meier analysis revealed no significant difference in overall survival (*p* = 0.928), local PFS (*p* = 0.339), or hepatic PFS among the cohorts (*p* = 0.946) (Fig. 2) when evaluated by underlying cause of HCC.

**Fig. 3:**
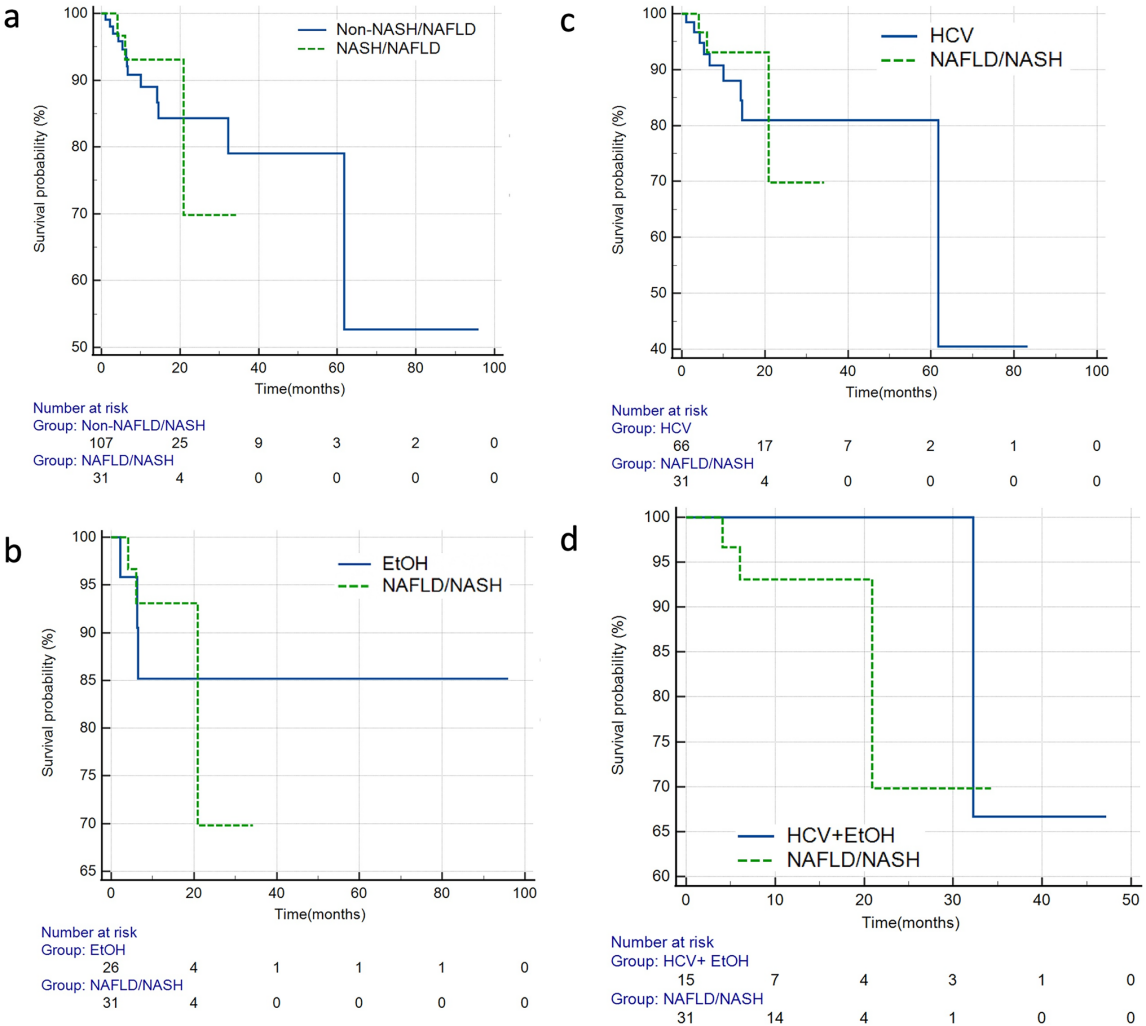
Kaplan Meier survival curves comparing overall survival for **a** NASH/NAFLD vs non-NASH/NAFLD (*p* = 0.886) **b** ALD vs NASH/NAFLD (*p* = 0.763) **c** HCV vs NASH/NAFLD and (*p* = 0.770) **d** ALD + HCV vs NASH/NAFLD (*p* = 0.403). Abbreviations: *NAFLD/NASH* non-alcoholic fatty liver disease/non-alcoholic steatohepatitis, *ALD* alcoholic liver disease, *HCV* hepatitis C virus, *HCV + ALD* hepatitis C virus + alcoholic liver disease.

After separating the entire cohort into non-NASH/NAFLD and NASH/NAFLD cohorts, there was no significant difference in OS (HR = 1.1, 95%CI: 0.32–3.79, *p* = 0.886) by log-rank analysis. Additionally, when discrete subgroup analyses comparing each etiology of HCC to the NASH/NAFLD cohort were performed, there was no significant difference in overall survival (Fig. 3, Table 2).

**Table 2:** Overall Survival of all patients in the cohort, stratified by etiology of HCC

Etiology	HR	95% C.I	*p* value
*All causes*	–	–	0.928
*NASH vs Non-NASH*	1.1	0.32–3.79	0.886
*NASH vs ALD*	0.78	0.16–3.92	0.763
*NASH vs HCV*	0.83	0.23–2.98	0.770
*NASH vs ALD/HCV*	2.48	0.3–20.8	0.403

Log-rank p values are reported

*NAFLD/NASH* (non-alcoholic fatty liver disease/non-alcoholic steatohepatitis), *ALD* (alcoholic liver disease), *HCV* (hepatitis C virus), *HCV + ALD* (hepatitis C virus + alcoholic liver disease)

**Fig. 4:**
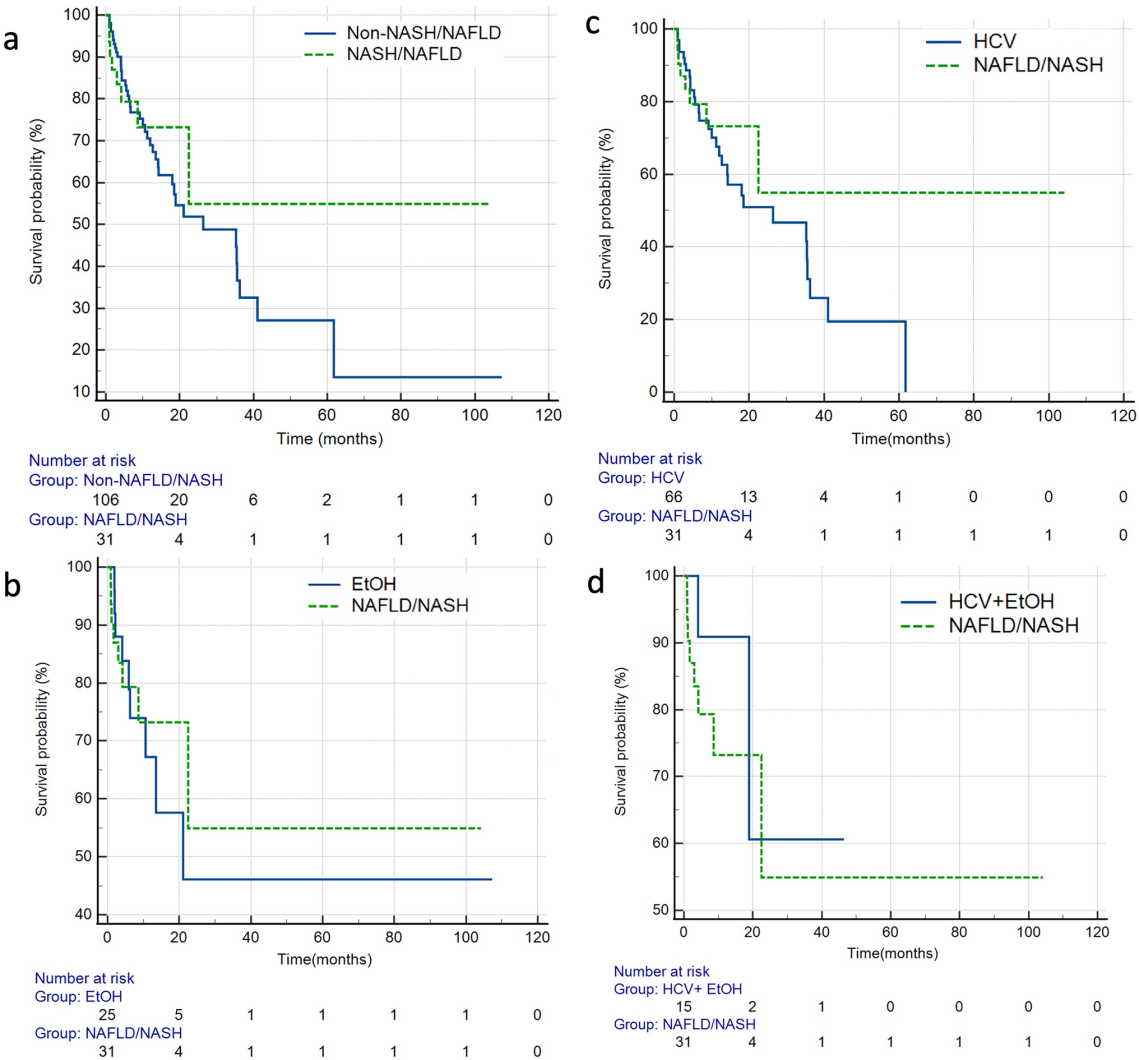
Kaplan Meier survival curves comparing local progression-free survival for **a** NASH/NAFLD vs non-NASH/NAFLD (*p* = 0.639) **b** ALD vs NASH/NAFLD (*p* = 0.705) **c** HCV vs NASH/NAFLD (*p* = 0.407) and **d** ALD + HCV vs NASH/NAFLD (*p* = 0.323). Abbreviations: *NAFLD/NASH* non-alcoholic fatty liver disease/non-alcoholic steatohepatitis, *ALD* alcoholic liver disease, *HCV* hepatitis C virus, *HCV+ALD* hepatitis C virus+alcoholic liver disease

When examining local PFS, there was no significant difference (HR = 1.2, 95%CI:0.58–2.44, *p* = 0.639) when the NASH/NAFLD cohort was compared to the non-NASH/NAFLD cohort (Fig. 4, Table 3) by log-rank analysis. Subgroup analyses comparing each etiology of HCC to the NASH/NAFLD cohort also did not demonstrate any significant difference in local PFS (Table 3).

**Table 3:** Local progression-free survival of all patients in the cohort, stratified by etiology

Etiology	HR	95% C.I	*p* value
*All causes*	–	–	0.339
*NASH vs Non-NASH*	1.2	0.58–2.44	0.639
*NASH vs ALD*	0.83	0.32–2.16	0.705
*NASH vs HCV*	0.74	0.36–1.52	0.407
*NASH vs ALD/HCV*	1.9	0.52–7.1	0.323

Log-rank p values are reported

*NAFLD/NASH* (non-alcoholic fatty liver disease/non-alcoholic steatohepatitis), *ALD* (alcoholic liver disease), *HCV* (hepatitis C virus), *HCV + ALD* (hepatitis C virus + alcoholic liver disease)

**Fig. 5:**
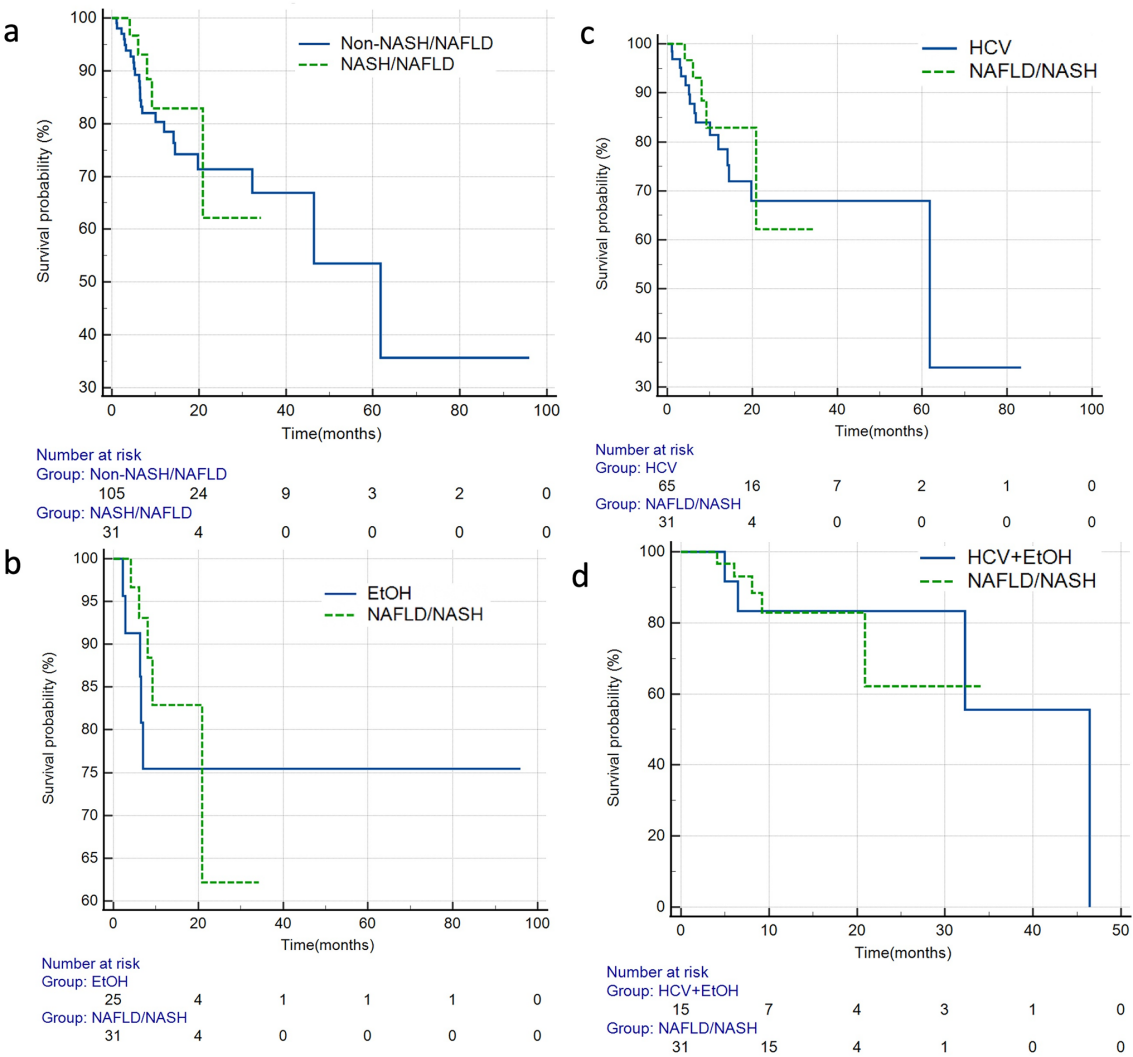
Kaplan Meier survival curves comparing hepatic progression-free survival for **a** NASH/NAFLD vs non-NASH/NAFLD (*p* = 0.595) **b** ALD vs NASH/NAFLD (*p* = 0.579) **c** HCV vs NASH/NAFLD (*p* = 0.592) and **d** ALD + HCV vs NASH/NAFLD (*p* = 0.995). Abbreviations: *NAFLD/NASH* non-alcoholic fatty liver disease/non-alcoholic steatohepatitis, *ALD* alcoholic liver disease, *HCV* hepatitis C virus, *HCV+ALD* hepatitis C virus+alcoholic liver disease

Finally, there was no significant difference in hepatic PFS when the NASH/NAFLD cohort was compared to the entire non-NASH/NAFLD cohort (HR = 1.3, 95%CI: 0.52–3.16, *p* = 0.595; Fig. 5, Table 4) by log-rank analysis. Similarly, when each cause of HCC was independently compared to the NASH/NAFLD cohort, there was no significant difference in hepatic PFS (Table 4).

**Table 4:** Hepatic progression-free survival of all patients in the cohort, stratified by etiology

Etiology	HR	95% C.I	*p* value
*All causes*	–	–	0.946
*NASH vs Non-NASH*	1.3	0.52–3.16	0.595
*NASH vs ALD*	0.7	0.2–2.47	0.579
*NASH vs HCV*	0.8	0.29–2.02	0.592
*NASH vs ALD/HCV*	1.0	0.22–4.48	0.995

Log-rank p values are reported

*NAFLD/NASH* (non-alcoholic fatty liver disease/non-alcoholic steatohepatitis), *ALD* (alcoholic liver disease), *HCV* (hepatitis C virus), *HCV + ALD* (hepatitis C virus + alcoholic liver disease)

### Post-TARE complications

There was no significant difference (*p* = 0.616) in post-radioembolization clinical toxicities as characterized by Common Terminology Criteria for Adverse Events (CTCAE) between each cohort. Two (2/52, 3.8%) patients in the HCV cohort suffered Grade 1 nausea. One (1/25, 4%) patient in the ALD cohort suffered Grade 1 nausea and two additional patients (2/25, 8%) suffered Grade 2 abdominal pain. Two (2/14, 14%) patients in the combined ALD/HCV cohort suffered Grade 1 abdominal pain. Five (5/30, 17%) patients in the NASH/NAFLD cohort suffered Grade 1 abdominal pain. In the HCV cohort, two patients suffered right common femoral access site complications, including one pseudoaneurysm requiring operative management.

### Radiologic response and tumor size

There was no difference in best overall radiologic response (progressive disease, stable disease, partial response, complete response) for the entire cohort when separated by underlying cause of HCC (*p* = 0.687, Table 5). A subgroup analysis comparing the best overall radiologic response rate for each cause of HCC to the NASH/NAFLD cohort did not demonstrate any significant difference (Table 6).

**Table 5:** Radiologic response rate by etiology of cirrhosis according to mRECIST 1.1 criteria

Radiologic response	
Entire cohort	
PD	19 (19/136, 14%)
SD	9 (9/136, 6.6%)
PR	35 (35/136, 25.7%)
CR	73 (73/136, 53.7%)
NAFLD/NASH	
PD	3 (3/31, 9.7%)
SD	2 (2/31, 6.5%)
PR	5 (5/31, 16.1%)
CR	21 (21/31, 67.7%)
ALD	
PD	3 (3/25, 12%)
SD	1 (1/25, 4%)
PR	7 (7/25, 28%)
CR	14 (14/25, 56%)
HCV	
PD	12 (12/65, 18.5%)
SD	6 (6/65, 9.2%)
PR	18 (18/65, 27.7%)
CR	29 (29/65, 44.6%)
HCV + ALD	
PD	1 (1/15, 6.7%)
SD	0 (0/15, 0%)
PR	5 (5/15, 33.3%)
CR	9 (9/15, 60%)

*NAFLD/NASH* (non-alcoholic fatty liver disease/non-alcoholic steatohepatitis), *ALD* (alcoholic liver disease), *HCV* (hepatitis C virus), *HCV + ALD* (hepatitis C virus + alcoholic liver disease, *PD* (progressive disease), *SD* (stable disease), *PR* (partial response), *CR* (complete response)

**Table 6:** Subgroup analysis comparing radiologic response rate, stratified by etiology of cirrhosis

Etiology	*p* value
*All causes*	0.687
*NASH vs ALD*	0.686
*NASH vs HCV*	0.233
*NASH vs ALD/HCV*	0.523

## Discussion

Although a prior study [23] did investigate the effectiveness of TARE in the NASH/NAFLD population; to the authors’ knowledge, this study is the first to compare the efficacy of TARE in the NASH/NAFLD population with other etiologies of HCC besides hepatitis B virus. Given the recent rise in NASH/NAFLD-induced HCC, its unique pathophysiology, and its historical lack of representation in literature regarding locoregional therapy, such investigations are needed.

These study’s results suggest that there is no significant difference in OS between the NASH/NAFLD cohort and the non-NASH/NAFLD cohort. There was also no significant difference in OS when various underlying etiologies of HCC were compared. While some recent studies have demonstrated comparatively better [16] and worsened [17] outcomes in NASH/NAFLD-induced HCC when treated with locoregional therapy and resection, additional studies [24] examining overall survival in the NASH/NAFLD population failed to demonstrate any difference in survival. These study’s results are also in agreement with a large, multinational retrospective analysis which did not demonstrate any difference in OS between NAFLD and HCV patients [25]. These differential results may be secondary to regional differences in the number of patients in the NASH/NAFLD cohort, as well as differences in local therapeutic practices. Finally, a combination of a lack of a standard consensus for screening NASH/NAFLD patients and complex socioeconomic factors such as poor access to healthcare in this population may play a factor in these varying results.

Furthermore, our results did not demonstrate a significant difference in hepatic or local PFS among all causes of HCC. This differs from a prior study [26] which demonstrated significantly better progression-free survival in NASH/NAFLD patients compared to patients with ALD/HCV, although patients in this study were treated with a combination of locoregional therapy including ablation and surgical resection. However, our study does concur with a prior study evaluating outcomes in NAFLD/NASH patients being treated with trans-arterial chemoembolization (TACE) [27]. Of note, tumorigenesis in NASH/NAFLD patients is incompletely understood; however, the effectiveness of TARE may be positively impacted by the over-abundant reactive oxygen species (ROS) known to be present in NAFLD/NASH due to chronically elevated fatty acid oxidation [28]. Prior research has demonstrated a synergistic therapeutic relationship between ionizing radiation such as that produced by Yttrium-90 microspheres [29], and increased levels of ROS [30]. However, further evaluation will be needed to determine what, if any, effect this has on clinical outcomes. Nonetheless, our data suggest that TARE is an appropriate form of locoregional therapy for early to intermediate stage NAFLD/NASH-induced HCC.

When examining population differences, our data do demonstrate a significant difference in tumor size and Child Pugh scores among the entire cohort. Additionally, there was a significant difference in the incidence of Type II diabetes within the NASH/NAFLD cohort compared to the non-NASH/NAFLD cohort. This is an expected finding given the association between metabolic syndrome and NASH/NAFLD as well as the differences in screening among these populations. This study is limited by its retrospective nature and relatively small sample size. Furthermore, the extremely low prevalence of HBV-induced cirrhosis in the local population may not be reflective of national trends. However, it does address a paucity of data on the clinical outcomes of TARE in this rapidly growing NASH/NAFLD patient population.

## Conclusion

This paper demonstrated no significant difference in OS, PFS, or local PFS in patients with NASH/NAFLD-induced HCC when compared to patients with other etiologies of HCC who underwent TARE. This indicates that although tumorigenesis is unique in NASH/NAFLD-induced HCC, TARE may be an appropriate form of locoregional therapy. Larger, ideally prospective multi-institutional cohorts are needed to further examine the use of TARE in the NASH/NAFLD-induced population.
